# Factors Associated with Disaster Preparedness Among Primary Healthcare Nurses in Makkah, Saudi Arabia: A Cross-Sectional Study

**DOI:** 10.3390/healthcare14152297

**Published:** 2026-07-29

**Authors:** Fahad M. Althobaiti, Manal M. Kabli

**Affiliations:** 1 Nursing Leadership and Education Department, Nursing College, Taif University, Taif 21974, Saudi Arabia; 2 Nursing Department, Al Noor Specialist Hospital, Makkah 24241, Saudi Arabia; mmkabli@moh.gov.sa

**Keywords:** disaster preparedness, primary healthcare nurses, mass gatherings, emergency readiness, Saudi Arabia, public health preparedness

## Abstract

**Background:** Primary healthcare centers are an essential component of disaster response systems, particularly in the Makkah Region of Saudi Arabia, where recurrent mass gatherings and environmental hazards increase the need for workforce readiness. Although nurses play a central role in frontline emergency response, evidence on disaster preparedness among primary healthcare nurses in Saudi Arabia remains limited. **Objective:** To assess the level of disaster preparedness among primary healthcare nurses in Makkah and examine demographic and professional factors associated with preparedness. **Methods:** A descriptive cross-sectional correlational study was conducted among 194 nurses working in primary healthcare centers in the Makkah Region. A multi-stage sampling approach was used in which 23 of 115 primary healthcare centers were randomly selected, followed by recruitment of eligible nurses through an online survey using non-probability methods. Disaster preparedness was measured using an adapted 68-item Disaster Preparedness Evaluation Tool covering biological and chemical preparedness, disaster response, family preparedness, disaster recovery, awareness and knowledge, communication and coordination, psychological preparedness, and training and participation. **Results:** Overall disaster preparedness was moderate to high (M = 3.84, SD = 0.75). The highest mean scores were observed for family preparedness (M = 4.56, SD = 1.11), biological and chemical preparedness (M = 4.32, SD = 0.95), and disaster response (M = 4.31, SD = 0.99), whereas training and participation had the lowest mean score (M = 2.65, SD = 0.54). Preparedness differed significantly by educational attainment, specialization, and years of experience, while no significant gender difference was observed. Correlations among preparedness dimensions were strong (r = 0.685–0.872), indicating that the measured preparedness domains were closely interrelated. **Conclusions:** Primary healthcare nurses in Makkah demonstrated generally favorable disaster preparedness; however, important gaps remain in training and participation, communication and coordination, and psychological preparedness. Strengthening simulation-based education, interdisciplinary drills, communication systems, and continuing professional development may improve frontline emergency readiness in primary care settings exposed to recurrent large-scale hazards.

## 1. Introduction

Disaster preparedness is a fundamental component of health system resilience because the continuity, safety, and effectiveness of healthcare delivery during emergencies depend on the readiness of frontline professionals and the organizations in which they work. The World Health Organization’s Health Emergency and Disaster Risk Management (Health EDRM) Framework emphasizes that preparedness is not limited to response capacity alone, but also includes prevention, readiness, recovery, coordination, risk communication, and the strengthening of community and system resilience [1]. Given the increasing frequency of disasters worldwide, nurses as key members of medical relief teams play a crucial role, and their level of preparedness greatly affects the effectiveness of disaster response [2]. Nurses, who frequently serve at the frontline of care, require well organized and high-quality training so they can manage critical incidents confidently and maintain resilience under pressure [3].

Primary healthcare services are especially important within disaster preparedness systems because they often represent the first point of contact for affected populations and play a major role in surveillance, triage, continuity of care, referral coordination, and health education during emergencies. For this reason, the preparedness of primary healthcare nurses should be viewed as a system-level issue rather than only an individual professional competency, particularly in settings where health services must respond to recurrent hazards and complex population demands [1].

In Saudi Arabia, disaster preparedness has growing strategic importance because the health sector must maintain readiness for infectious disease threats, environmental hazards, and crowd-related emergencies. This need is especially pronounced in the Makkah Region, which experiences recurrent large-scale population movements associated with Hajj and Umrah and therefore faces heightened risks related to overcrowding, infectious diseases, extreme heat, and other emergency conditions [4,5].

Makkah represents a uniquely important context for disaster preparedness research because the volume and concentration of pilgrims can place substantial pressure on healthcare resources and emergency response systems. Saudi Arabia has invested in mass-gathering health planning and capacity building, including the development of national expertise and institutional frameworks to strengthen readiness and protect population health during large-scale events [5].

Nurses constitute the largest segment of the healthcare workforce and are central to disaster management through their roles in triage, patient stabilization, communication, coordination, public education, and continuity of care. Previous research has shown that disaster preparedness among nurses is influenced by multiple factors, including prior training, clinical exposure, disaster drills, role clarity, organizational support, and access to policies and operational guidance [1,4].

Preparedness is also multidimensional. In addition to technical competencies, effective disaster response requires communication and coordination skills, psychological readiness, and the ability to function within interdisciplinary systems under pressure. The Health EDRM framework highlights the need for a whole-system and multisectoral approach, indicating that individual preparedness is inseparable from the organizational and policy environments in which healthcare workers operate [1].

Despite growing international attention to disaster preparedness, important evidence gaps remain in relation to primary healthcare nurses, particularly in mass-gathering settings. Much of the existing literature has focused on hospital-based nurses, emergency departments, or general healthcare workers, while fewer studies have examined the preparedness of nurses working in primary healthcare settings where community-facing response functions are especially important. This gap is particularly relevant in Saudi Arabia because preparedness demands in primary healthcare extend beyond acute clinical response to include referral coordination, communication with families and communities, and sustained service continuity during disruptive events. Evidence from Saudi mass-gathering and healthcare settings suggests that training, drills, and perceived readiness may vary across institutions, reinforcing the need for context-specific assessment within primary healthcare services [4].

Although previous studies have identified the importance of demographic, educational, and organizational influences on disaster preparedness, fewer studies have examined how these factors relate to multiple preparedness domains among primary healthcare nurses in the Makkah Region. In addition, limited evidence is available on whether the dimensional structure of preparedness tools adequately reflects the way preparedness is experienced in this frontline setting. Addressing these gaps is important for informing workforce development, strengthening primary care emergency readiness, and supporting disaster risk reduction strategies in Saudi Arabia and comparable mass-gathering environments [1,5].

Therefore, this study aimed to assess the level of disaster preparedness among primary healthcare nurses in Makkah, Saudi Arabia, examine demographic and professional factors associated with preparedness, and explore relationships among preparedness dimensions.

## 2. Methods

### 2.1. Research Design

This study used a descriptive cross-sectional correlational design to assess disaster preparedness among primary healthcare nurses and examine associations with selected demographic and professional characteristics.

### 2.2. Setting

The study was conducted in primary healthcare centers (PHCCs) located across the Makkah Region, Saudi Arabia. This setting was selected because the region is exposed to recurrent public health and disaster risks, including crowd-related incidents, infectious disease threats, flooding, and extreme temperatures, and because PHCCs serve as frontline health facilities for both resident and mass-gathering populations.

### 2.3. Participants and Sampling

A multi-stage sampling approach was used. In the first stage, 20% of all PHCCs in the Makkah Region were randomly selected from the total of 115 centers, resulting in 23 participating PHCCs. In the second stage, convenience and snowball sampling were used to recruit eligible nurses within the selected centers, which means that, although centers were randomly chosen, the nurse sample at each center was obtained using non-probability methods and may therefore be subject to selection bias.

Sample size was estimated a priori using G*Power version 3.1.9.7 for a one-way analysis of variance (ANOVA), with an alpha level of 0.05, statistical power of 0.80, and a medium effect size (f = 0.30), consistent with effect sizes commonly observed in disaster preparedness studies. This calculation indicated a minimum required sample of 64 participants. To improve the precision of estimates, support subgroup analyses based on educational qualification, years of experience, and nursing specialty, and account for potential non-response or incomplete questionnaires, the recruitment target was set higher than this minimum. Ultimately, 194 nurses completed the survey and were included in the final analysis.

### 2.4. Research Instrument

Data were collected using two instruments. The first was a researcher-developed demographic and professional information form that captured participants’ sex, age, highest educational qualification, nursing specialty, and years of professional experience.

The second instrument was an adapted version of the Disaster Preparedness Evaluation Tool (DPET) [6], derived from the original instrument and its subsequent revisions for use in nursing populations. The adapted questionnaire used in the present study comprised 68 items rated on a 5-point Likert scale ranging from 1 (strongly disagree) to 5 (strongly agree), with higher scores indicating greater perceived disaster preparedness. The instrument assessed eight domains: biological and chemical preparedness, disaster response, family preparedness, disaster recovery, awareness and knowledge, communication and coordination, psychological preparedness, and training and participation.

Before implementation, the instrument was reviewed for suitability in the Saudi primary healthcare context. An expert panel composed of specialists in disaster nursing, community health nursing, and nursing education reviewed the adapted DPET for content relevance, clarity, cultural appropriateness, and contextual fit for PHCC practices in Makkah. Minor linguistic and contextual modifications were made to improve readability and ensure alignment with local terminology, disaster-response structures, and the roles of nurses working in primary healthcare settings. No major changes were made to the conceptual meaning of the items or to the underlying domain structure. A pilot test was also conducted with nurses to assess item clarity, acceptability, and completion time; feedback from the pilot resulted in only minor wording refinements and no substantive changes to the content or scoring of the instrument.

The decision to use the DPET as an adapted measure was supported by previous validation studies in nursing populations, which reported acceptable content validity, factor structure, and internal consistency after processes such as translation, expert review, pilot testing, and exploratory or confirmatory factor analysis. In the present study, internal consistency was high, with a Cronbach’s alpha of 0.94 for the overall scale. Subscale reliability coefficients were 0.88 for biological and chemical preparedness, 0.90 for disaster response, 0.84 for family preparedness, 0.82 for disaster recovery, 0.86 for awareness and knowledge, 0.89 for communication and coordination, 0.78 for psychological preparedness, and 0.81 for training and participation, indicating acceptable to excellent reliability across domains in this sample.

### 2.5. Data Collection Procedure

Data were collected using a web-based questionnaire administered to eligible nurses working in the selected PHCCs. Eligible participants received an invitation link through their workplace communication channels and accessed the survey at a time convenient to them.

Before accessing the questionnaire, participants were presented with an electronic informed consent form. The consent form described the purpose of the study, the procedures involved, the voluntary nature of participation, the right to withdraw at any time without penalty, and any potential risks or discomfort, as well as the measures taken to protect confidentiality.

Only participants who indicated their consent electronically were able to proceed to the survey. To enhance data integrity, submitted responses were reviewed during data cleaning; incomplete or clearly inconsistent questionnaires were excluded, and Z score-based screening was applied prior to statistical analysis to identify outliers or implausible values. During data cleaning, continuous preparedness scores were examined for potential outliers using Z-score screening, with absolute Z-values greater than 3.29 flagged for review. All flagged cases were checked against the original records, and none showed evidence of data-entry error or implausible values, so no participants were excluded on the basis of this procedure. The final analytic sample therefore comprised 194 nurses.

All completed questionnaires were anonymized and stored in password-protected electronic systems with restricted access. Data were maintained using encryption and data protection safeguards consistent with Ministry of Health requirements and institutional data security standards, and no directly identifying information was retained.

### 2.6. Data Analysis

Data were analyzed using IBM SPSS Statistics version 26.0. To address the study aims, the analysis was structured to describe overall and domain-specific disaster preparedness, compare preparedness across demographic and professional groups, and examine relationships among preparedness dimensions. Descriptive statistics, including frequencies, percentages, means, and standard deviations, were used to summarize participants’ demographic and professional characteristics and to describe disaster preparedness scores across the measured domains. An independent-samples *t* test was used to compare preparedness scores based on gender, whereas one-way analysis of variance (ANOVA) was used to assess differences according to educational attainment, grouped nursing specialty, and years of nursing experience. When the homogeneity-of-variance assumption was violated, Welch’s ANOVA was adopted as the primary omnibus test with Games–Howell post hoc comparisons; when homogeneity of variance was satisfied, Tukey’s honestly significant difference test was applied. Post hoc procedures were applied after each significant omnibus test for educational qualification, years of experience, and grouped nursing specialty to identify specific group differences. Pearson product-moment correlation coefficients were computed to examine relationships among the preparedness domains and between each domain and overall disaster preparedness. To identify factors independently associated with overall disaster preparedness, hierarchical multiple linear regression was conducted with overall preparedness entered as the dependent variable. In the first step, demographic and professional characteristics (gender, educational qualification, years of experience, and grouped nursing specialty) were entered as predictors; in the second step, previous exposure to a real disaster and disaster-preparedness training during nursing education were added. Model fit was evaluated using R, $R^{2}$, adjusted $R^{2}$, change in $R^{2}$, F statistics, and the standard error of the estimate, and multicollinearity was assessed using tolerance and variance inflation factor values. All statistical tests were interpreted at a significance level of *p* < 0.05. Moreover, because the study used a descriptive cross-sectional design, all analyses were interpreted as indicating associations between variables rather than causal relationships.

### 2.7. Ethical Considerations

Ethical approval for this study was obtained from the Institutional Review Board (IRB) of the Makkah healthcare cluster (H-02-K-076-1124-1223). Participation was entirely voluntary, and electronic informed consent was obtained from all respondents before they accessed the questionnaire. The electronic consent form provided information about the purpose of the study, study procedures, potential risks and benefits, confidentiality protections, and the participants’ right to decline or withdraw from participation at any time without penalty. No directly identifying information was collected, and all responses were treated confidentially throughout the study process. Completed survey data were anonymized and stored in password-protected electronic systems with restricted access, in accordance with institutional requirements and applicable data protection standards.

## 3. Results

A total of 194 nurses working in primary healthcare centers in the Makkah Region were included in the analysis. The sample was predominantly mid-career, with most participants aged 41–50 years and holding diploma or master’s qualifications; general nursing was the most common specialty, and most nurses had more than 10 years of experience. Disaster-related activities were common: the large majority reported regular workplace drills, perceived these drills as beneficial, and had received disaster-preparedness training during their studies, with most also indicating regular participation in continuing education, drills, and peer evaluations related to disaster preparedness. Detailed demographic, professional, and training characteristics are presented in Table 1.

Disaster preparedness scores indicated moderate-to-high overall readiness, with a mean of 3.84 (SD = 0.75) on the 5-point scale. Family preparedness, biological and chemical preparedness, and disaster response showed the highest means, whereas training and participation had the lowest mean (M = 2.65, SD = 0.54), substantially below other domains and near the lower boundary of the moderate range. This pattern suggests that nurses’ generally favorable perceptions of readiness are not matched by sustained engagement in structured training and participation activities. Full domain scores and interpretations are shown in Table 2.

Overall disaster preparedness did not differ by gender, with male and female nurses reporting similar preparedness. Preparedness was higher among nurses with postgraduate education, in emergency and family/community specialties, and generally more years of experience; full group means are shown in Table 3.

Strong positive correlations were observed among preparedness dimensions, particularly among awareness and knowledge, communication and coordination, and disaster response, indicating that information, communication, and response capabilities are closely interconnected. The weakest, though still substantial, correlation was between psychological preparedness and family preparedness, suggesting that emotional readiness may be somewhat more distinct from family-related planning than other domains. Intercorrelations are presented in Table 4.

Post hoc comparisons indicated that nurses with postgraduate qualifications scored consistently higher than those with bachelor’s degrees across all domains and on overall readiness, while differences between postgraduate and diploma holders were more limited. For years of experience, readiness tended to peak among nurses with 6–10 years of experience and then decline in the most senior groups, although this pattern should be interpreted cautiously given the cross-sectional design. Key post hoc contrasts are reported in Table 5.

In the hierarchical regression model, demographic and professional characteristics explained a modest proportion of variance in overall readiness, which increased when previous disaster exposure and disaster-preparedness training during nursing education were added. Training during nursing education emerged as the strongest independent predictor of readiness, with nurses who had received such training scoring notably higher after adjustment for other variables, while the 6–10-year experience group also showed a small but statistically significant advantage over the least experienced group. Other predictors, including gender, educational level, specialty, longer experience categories, and prior disaster exposure, were not significant independent predictors in the final model. Model parameters and diagnostics are summarized in Table 6.

## 4. Discussion

This study examined disaster preparedness among primary healthcare nurses in the Makkah Region and found that overall preparedness was moderate to high, with meaningful variation across domains. Nurses perceived themselves as particularly well-prepared in family preparedness, biological and chemical preparedness, and disaster response, reflecting strong perceived technical and personal readiness for many aspects of disaster response. However, the training and participation domain was notably lower than all other domains, with a mean score close to the lower boundary of the moderate range. This discrepancy indicates that perceived readiness is not fully supported by sustained participation in formal training, drills, and continuing education activities, and it represents a critical gap in the preparedness profile of primary healthcare nurses.

The very high correlations among several preparedness domains suggest that, although these domains are theoretically distinct, they may represent closely related facets of a broader underlying preparedness construct rather than fully independent dimensions. This possibility of conceptual overlap should be considered when interpreting domain-specific findings, and future studies should use exploratory and confirmatory factor analysis to clarify the dimensional structure of the adapted DPET. At the same time, disaster preparedness within primary healthcare centers is inherently multidisciplinary and depends on the coordinated efforts of physicians, nurses, emergency medical services personnel, administrators, and other healthcare workers. Consequently, the present findings should be interpreted as characterizing the nursing component of PHCC preparedness rather than the full, team-based capability of primary healthcare services, and future research should adopt a multi-professional design to capture how the preparedness of different cadres jointly shapes overall system readiness during emergencies. Furthermore, these findings should be understood as associative rather than causal, as the cross-sectional design does not allow for temporal ordering or control of unmeasured confounding.

The high scores in family preparedness, biological and chemical preparedness, and disaster response may be influenced by the distinctive operational and spiritual context of Makkah, where nurses routinely work in an environment exposed to mass gatherings and a wide range of hazards [7]. In such settings, disaster response is often perceived as both a professional obligation and a moral responsibility, which can enhance vigilance and commitment during emergencies [8,9]. Similar to findings from Hajj-related studies that have documented high perceived competencies among healthcare personnel involved in repeated mass-gathering operations [4,7], the nurses in this study reported consistently high preparedness in several operational domains.

At the same time, the relatively low score for training and participation highlights a gap between perceived readiness and the extent of structured, ongoing preparedness activities. Prior work in Saudi Arabia has identified inconsistent disaster curricula, limited access to structured training, and unclear role expectations as barriers to sustained preparedness among nurses [10,11]. These observations are consistent with the World Health Organization’s Health Emergency and Disaster Risk Management (Health EDRM) framework, which emphasizes institutionalized training, clear communication pathways, and coordinated systems as core components of effective preparedness [1]. The current findings suggest that although primary healthcare nurses in Makkah possess strong technical and personal readiness, they may benefit from more regular, competency-based simulation, interprofessional drills, and structured communication protocols embedded within routine practice.

The absence of significant gender differences in preparedness aligns with studies indicating that disaster preparedness is shaped more by access to training, organizational culture, and institutional support than by sex [12,13]. In contrast, significant differences by educational attainment, clinical specialization, and years of experience indicate that professional development and exposure play an important role in shaping preparedness. Nurses with higher degrees and greater experience reported higher levels of preparedness in this study, supporting the view that advanced education and cumulative clinical exposure contribute to enhanced decision-making, technical skills, and confidence in managing disasters [14,15].

Specialization also appeared to influence preparedness. Nurses working in emergency and family or community nursing reported higher preparedness than those in general nursing, which is consistent with studies showing that nurses in emergency and community-facing roles, who have more frequent exposure to acute events and public health functions, tend to report higher disaster preparedness [16,17]. These patterns support a tiered preparedness strategy that leverages both formal education and role-specific experience when designing disaster training and workforce development programs.

The observed associations between preparedness and years of experience are also consistent with work suggesting that more experienced nurses bring deep clinical judgment and crisis-tested confidence, while younger nurses often possess greater familiarity with digital systems, simulation technology, and coordination tools [18]. Blended learning models that combine mentorship from experienced practitioners with simulation-based and technology-enhanced training for less experienced staff may therefore help create a more uniformly prepared workforce. National and institutional policies that ensure regular participation in continuing professional development—including disaster preparedness, interprofessional collaboration, and leadership training—are likely to be particularly important in sustaining these gains [14,15].

The strong positive correlations among preparedness dimensions observed in this study indicate that disaster preparedness in primary healthcare is multidimensional and interdependent rather than composed of isolated competencies. The close relationships among awareness and knowledge, communication and coordination, and disaster response suggest that effective performance during emergencies depends on the integration of cognitive understanding, clear information flow, and coordinated teamwork [12,16]. When knowledge is not effectively communicated, or when coordination mechanisms are weak, technical competence alone may not translate into effective responses.

The comparatively weaker correlation between psychological preparedness and family preparedness, although still substantial, may indicate that personal contingency planning does not automatically translate into emotional resilience in prolonged or complex crises. This finding reinforces the importance of addressing psychological readiness and stress management explicitly within preparedness programs. Other studies have similarly emphasized that the absence of coping strategies and psychological support can undermine the benefits of strong technical skills and team coordination during disasters [8,19].

From an international perspective, Feng et al. synthesized global evidence on nurses’ disaster preparedness in a systematic review and meta-analysis, identifying disaster-response experience, prior disaster training, gender, age, professional rank, educational level, years of practice, hospital type, and work department as key factors associated with preparedness. Their review showed that nurses typically exhibit only moderate levels of disaster preparedness, underscoring the need for management to design tailored emergency plans and training programs that account for diverse staff characteristics. Feng et al. further noted that, worldwide, nurses’ disaster preparedness remains suboptimal and that meaningful improvement will require coordinated action by national authorities, professional organizations, nursing schools, disaster nursing scholars, healthcare institutions, and nurses themselves. They advocated embedding disaster preparedness into national development and budgetary priorities, with a strong emphasis on proactive readiness rather than reliance on post-disaster recovery [20]. Consistent with these findings, Lou et al. reported that nurses’ overall disaster preparedness was moderate, with notable gaps in practical skills and management capabilities, and that emergency department nurses without prior disaster response experience or training demonstrated particularly low preparedness levels [2]. Building on this body of evidence, Golpinar et al. emphasized that strengthening the disaster preparedness of healthcare personnel should be treated as a priority for effective disaster management [21].

### Practice Implications

The findings point to several practical implications for primary healthcare systems in disaster-prone and mass-gathering settings. First, the low scores in training and participation justify the introduction or strengthening of mandatory, competency-based disaster training as part of continuing professional development for nurses. This recommendation is consistent with prior calls for structured, recurrent preparedness training that includes drills, scenario-based simulations, and formal evaluation of competencies [13,22]. Such training should be tailored to the specific hazards and operational realities of Makkah and should incorporate structured debriefings to identify system-level gaps.

Second, institutions should enhance interprofessional drills and communication protocols that link primary healthcare centers with hospitals, emergency medical services, and public health agencies. Clear communication pathways, defined roles, and coordinated team structures are likely to improve both confidence and performance during real events, in line with recommendations from previous work on disaster nursing and health system preparedness [11,17]. Third, preparedness initiatives should explicitly address psychological readiness and resilience by incorporating content on stress management, coping strategies, and post-event recovery, recognizing that emotional endurance is a critical complement to technical skills [8,9].

Finally, policies and workplace supports that promote family readiness—such as access to information, resources, and contingency planning tools for nurses’ families—may reduce personal barriers to participation and allow nurses to remain fully engaged in their professional roles during emergencies. This is consistent with evidence that family concerns can influence healthcare workers’ willingness to respond in disaster scenarios [23,24]. Additionally, at the national primary healthcare level, disaster preparedness should be institutionalized through standardized curricula, simulation-based and interprofessional drills, and integration of disaster-readiness competencies into continuing professional development, workforce planning, and support systems (including psychological support and communication infrastructure) for PHCC staff.

## 5. Strengths and Limitations

This study contributes to the limited literature on disaster preparedness among primary healthcare nurses in a high-risk mass-gathering region and uses a multidimensional instrument to capture both technical and psychosocial aspects of readiness [14,15]. The sample included nurses with a broad range of educational backgrounds, specialties, and years of experience, allowing for exploration of important professional correlates of preparedness. However, the cross-sectional design and reliance on self-reported data limit causal inference and introduce the possibility of social desirability bias. Non-probability sampling of nurses within randomly selected centers may also limit generalizability beyond similar primary healthcare contexts. Future research could incorporate objective performance measures, longitudinal designs, or mixed-methods approaches to better capture how preparedness evolves over time and in response to specific interventions [16,19].

A further limitation is that the study did not perform exploratory or confirmatory factor analysis of the adapted DPET. Given the high inter-domain correlations, future research should examine the instrument’s factor structure and discriminant validity more formally. In addition, the study focused exclusively on nurses. Disaster preparedness in primary healthcare centers is inherently multidisciplinary, and the preparedness of physicians, EMS personnel, administrative staff, and other healthcare workers was not assessed. Consequently, convenience and snowball sampling of nurses within selected centers may have introduced selection bias and limits the generalizability of the findings even within the nursing workforce; future studies should include multiple professional groups to provide a more comprehensive picture of primary healthcare disaster preparedness.

The study did not assess participants’ previous involvement in Hajj, Umrah, or other mass-gathering responses. Such experience may substantially influence preparedness, so future studies should include specific measures of mass-gathering exposure. Finally, because of the cross-sectional and observational design, the observed relationships should be interpreted as associations rather than causal effects.

## 6. Conclusions

Primary healthcare nurses in the Makkah Region reported generally favorable levels of disaster preparedness, with stronger scores in family preparedness, biological and chemical preparedness, and disaster response than in other domains. However, the comparatively low score for training and participation highlights an important weakness, suggesting that perceived readiness is not consistently supported by sustained engagement in structured disaster education and drills. Preparedness did not differ meaningfully by gender, whereas higher levels were observed among nurses with stronger educational and professional profiles in unadjusted analyses. In the multivariable model, disaster-preparedness training during nursing education was the strongest independent factor associated with overall readiness, and nurses with 6–10 years of experience showed higher adjusted readiness than those with 1–5 years. These factors were associated with higher readiness; however, the cross-sectional design precludes causal conclusions.

## 7. Recommendations

The findings support the need for institutionalized and ongoing preparedness strategies within primary healthcare settings in Makkah, including competency-based simulation, regular interprofessional drills, structured continuing professional development, and psychological preparedness support, with particular emphasis on strengthening the training and participation domain. Enhancing these areas may improve the operational readiness of primary healthcare nurses and bolster the capacity of PHCCs to respond effectively to emergencies affecting both resident populations and mass-gathering communities in the region.

## Figures and Tables

**Table 1:** Participant characteristics (n = 194).

Category	Characteristic	Count	Percentage (%)
Gender	Female	77	39.7
	Male	117	60.35
Age	22–30	31	16.0
	31–40	64	33.0
	41–50	79	40.7
	51 and above	20	10.3
Highest Educational Qualification	Diploma degree	97	50.0
	Bachelor’s degree	40	20.6
	Master’s degree	54	27.8
	Doctoral degree	3	1.5
Current Specialization	General Nursing	99	51.0
	Emergency Nursing	18	9.3
	Family and Community Nursing	38	19.6
	Medical surgical nursing	1	0.5
	Dialysis Nurse	1	0.5
	Midwife	2	1.0
	Pediatric Nursing	24	12.4
	Mental Health Nursing	11	5.7
Years of Experience	1–5 years	27	13.9
	6–10 years	26	13.4
	11–15 years	65	33.5
	16–20 years	55	28.4
	More than 20 years	21	10.8
**Training and participation related to emergency disaster preparedness**			
Are disaster drills conducted regularly at your workplace?	No	15	7.7%
	Yes	179	92.3%
Do you find the disaster drills beneficial for preparedness?	No	11	5.7%
	Yes	183	94.3%
Have you received disaster preparedness training during your studies?	No	77	39.7%
	Yes	117	60.3%
I regularly participate in continuing education activities, seminars, or conferences related to disaster preparedness.	Disagree	21	10.8%
	Somewhat Disagree	4	2.1%
	Somewhat Agree	73	37.6%
	Agree	76	39.2%
	Strongly Agree	20	10.3%
I actively participate in disaster drills or simulation exercises at my workplace.	Disagree	24	12.4%
	Somewhat Disagree	5	2.6%
	Somewhat Agree	56	28.9%
	Agree	84	43.3%
	Strongly Agree	25	12.9%
I regularly participate in peer evaluations to improve disaster preparedness skills.	Disagree	19	9.8%
	Somewhat Disagree	2	1.0%
	Somewhat Agree	96	49.5%
	Agree	71	36.6%
	Strongly Agree	6	3.1%

**Table 2:** Disaster preparedness levels.

Preparedness Dimension	Mean (M)	Standard Deviation (SD)	Interpretation
Family Preparedness	4.56	1.11	Very High
Biological and Chemical Preparedness	4.32	0.95	High
Disaster Response	4.31	0.99	High
Disaster Recovery	3.87	0.91	Moderate to High
Awareness and Knowledge	3.75	0.89	High
Communication and coordination	3.55	0.90	High
Psychological Preparedness	3.42	0.68	High
Training and Participation	2.65	0.54	Low
Overall Preparedness	3.84	0.75	Moderate to High

Note: Interpretation was based on a 5-point Likert scale, where 1.00–1.80 = Very Low, 1.81–2.60 = Low, 2.61–3.40 = Moderate, 3.41–4.20 = High, and 4.21–5.00 = Very High.

**Table 3:** Overall disaster preparedness by demographic and professional characteristics.

Predictor	Group/Level	Mean (M)	SD	Test Statistic	df	*p*
Gender	Male	4.33	0.92	t = 0.369	192	0.713
	Female	4.31	0.93			
Educational attainment	Diploma degree	4.20	0.95	F = 5.886	3, 190	0.001
	Bachelor’s degree	4.45	0.87			
	Master’s degree	4.41	0.90			
	Doctoral degree	4.56	0.88			
Current specialization	General nursing	4.31	0.94	F = 5.900	3, 190	0.001
	Emergency nursing	4.59	0.81			
	Family & community nursing	4.45	0.87			
	Other	4.42	0.86			
Years of experience	1–5 years	4.18	0.98	F = 4.328	4, 189	0.002
	6–10 years	4.23	0.91			
	11–15 years	4.43	0.90			
	16–20 years	4.45	0.89			
	>20 years	4.47	0.89			

**Table 4:** Relationships among preparedness dimensions.

Factor	1	2	3	4	5	6	7
1. Biological & Chemical	1	0.869	0.725	0.812	0.705	0.809	0.770
2. Disaster Response		1	0.724	0.792	0.747	0.838	0.783
3. Family Preparation			1	0.770	0.700	0.685	0.765
4. Knowledge & Awareness				1	0.769	0.782	0.872
5. Training & Participation					1	0.713	0.743
6. Psychological & Emotional						1	0.786
7. Communication & Coordination							1

**Table 5:** Post hoc comparisons for overall disaster preparedness and preparedness domains.

Outcome	Significant Comparison	Mean Difference	*p*-Value	95% Confidence Interval
Biological and Chemical Preparedness	Postgraduate > Bachelor’s	0.660	<0.001	[0.353, 0.967]
Disaster Response	Postgraduate > Bachelor’s	0.592	<0.001	[0.279, 0.906]
Family Preparation	Postgraduate > Bachelor’s	0.594	0.001	[0.217, 0.971]
Knowledge and Awareness	Postgraduate > Bachelor’s	0.623	<0.001	[0.301, 0.946]
Training and Participation	Postgraduate > Bachelor’s	0.525	0.002	[0.159, 0.890]
Psychological and Emotional Preparedness	Postgraduate > Bachelor’s	0.607	<0.001	[0.278, 0.936]
Communication and Coordination	Postgraduate > Bachelor’s	0.659	<0.001	[0.320, 0.998]
Communication and Coordination	Postgraduate > Diploma	0.442	0.048	[0.003, 0.880]
Overall Readiness	Postgraduate > Bachelor’s	0.615	<0.001	[0.314, 0.915]
Biological and Chemical Preparedness	6–10 years > More than 21 years	0.777	0.020	—
Disaster Response	6–10 years > 16–20 years	0.619	0.015	—
Disaster Response	6–10 years > More than 21 years	0.803	0.040	—
Knowledge and Awareness	6–10 years > 16–20 years	0.627	0.012	—
Knowledge and Awareness	6–10 years > More than 21 years	0.835	0.023	—
Training and Participation	6–10 years > More than 21 years	0.813	0.022	—
Psychological and Emotional Preparedness	6–10 years > 1–5 years	0.693	0.041	—
Psychological and Emotional Preparedness	6–10 years > 16–20 years	0.679	0.004	—
Psychological and Emotional Preparedness	6–10 years > More than 21 years	0.872	0.009	—
Communication and Coordination	6–10 years > 16–20 years	0.570	0.025	—
Overall Readiness	6–10 years > 16–20 years	0.587	0.012	—
Overall Readiness	6–10 years > More than 21 years	0.804	0.020	—

**Table 6:** Hierarchical multiple linear regression predicting overall disaster preparedness.

Predictor	B	SE	β	t	*p*-Value	95% Confidence Interval	VIF
Constant	3.377	0.367	—	9.210	<0.001	[2.654, 4.101]	—
Female vs. male	0.021	0.118	0.011	0.178	0.859	[−0.213, 0.255]	1.055
Diploma vs. bachelor’s	0.107	0.167	0.048	0.642	0.522	[−0.222, 0.437]	1.433
Postgraduate vs. bachelor’s	0.293	0.188	0.148	1.558	0.121	[−0.078, 0.664]	2.302
Experience 6–10 vs. 1–5 years	0.487	0.246	0.204	1.979	0.049	[0.002, 0.973]	2.686
Experience 11–15 vs. 1–5 years	0.194	0.229	0.102	0.847	0.398	[−0.258, 0.647]	3.681
Experience 16–20 vs. 1–5 years	0.315	0.234	0.158	1.347	0.180	[−0.147, 0.778]	3.503
Experience > 21 vs. 1–5 years	0.382	0.279	0.135	1.370	0.172	[−0.169, 0.933]	2.462
Emergency/acute care vs. general nursing	0.243	0.238	0.082	1.022	0.308	[−0.226, 0.713]	1.646
Family/community vs. general nursing	0.129	0.216	0.057	0.600	0.549	[−0.296, 0.555]	2.300
Maternal/child vs. general nursing	0.190	0.229	0.072	0.830	0.408	[−0.262, 0.643]	1.920
Mental health vs. general nursing	0.466	0.298	0.120	1.564	0.119	[−0.122, 1.054]	1.491
Previous real-disaster exposure	−0.051	0.306	−0.011	−0.165	0.869	[−0.654, 0.553]	1.163
Training during nursing education	0.717	0.146	0.391	4.904	<0.001	[0.429, 1.006]	1.610

## Data Availability

The data presented in this study are available on request from the corresponding author. The data are not publicly available due to privacy and ethical restrictions.
